# Predictive value of adiposity measures and lipid-related indices for metabolic syndrome in Chinese elderly people at high risk of stroke

**DOI:** 10.1371/journal.pone.0328275

**Published:** 2025-08-12

**Authors:** Ran Chen, Jun Li, Daikun He, Shengqiong Pan

**Affiliations:** 1 Department of General Practice, Jinshan Hospital, Fudan University, Shanghai, China; 2 Department of General Practice, Zhujing Community Health Service Center, Jinshan District, Shanghai, China; 3 Department of General Practice, Lvxiang town Community Health Service Center, Jinshan District, Shanghai, China; University of Diyala College of Medicine, IRAQ

## Abstract

This study aims to explore the predictive value of adiposity measures and lipid-related indices for metabolic syndrome (MetS) in Chinese elderly people at high risk of stroke. A total of 7,693 community-dwelling individuals aged 60 years and older in Shanghai were included. Through ROC curve analysis, it was found that indices such as triglyceride glucose-waist circumference (TyG-WC), triglyceride glucose-waist-to-height-ratio (TyG-WHtR), and Lipid accumulation product (LAP) showed excellent performance in predicting MetS, with TyG-WC being the strongest predictor in both males (AUC = 0.90) and females (AUC = 0.90). These indices integrate information on both lipid metabolism and body fat distribution, allowing for more accurate identification of MetS. The study also revealed that ABSI had poor predictive performance. These findings suggest that the combined use of these indices may help identify MetS earlier and more accurately in elderly people at high risk of stroke, providing a basis for clinical intervention. Future research should validate the generalizability of these indicators in more diverse populations and explore their potential integration into clinical workflows.

## Introduction

Metabolic syndrome (MetS) is a common condition in the elderly, characterized by the presence of three or more of the following risk factors: abdominal obesity, hypertension, glucose intolerance, and dyslipidemia [1]. These risk factors also predispose individuals to atherosclerotic cardiovascular disease and type 2 diabetes, which can further lead to severe complications such as atherosclerosis, cerebral infarction, and myocardial infarction [2]. Alarmingly, the prevalence of MetS remains high and is continuing to rise in both developed and developing countries [3].A study conducted in China in 2020 indicated that 33.38% of the Chinese population was affected by MetS (95% CI 32.42–34.34%) [4]. MetS has now been widely recognized as a significant clinical and public health issue, necessitating early detection to prevent severe complications [5].

Stroke is a significant global health issue, ranking as the second leading cause of death and the primary cause of long-term disability [6]. In China, the prevalence of stroke continues to rise, imposing a substantial burden on the healthcare system [7]. MetS is a major risk factor for cardiovascular diseases and is associated with an increased risk of stroke, as well as a heightened likelihood of stroke recurrence [8]. Therefore, it is crucial to distinguish between patients with and without MetS within the high-risk stroke population. By addressing MetS, we can ultimately reduce its prevalence and, consequently, lower the risk of stroke. In this study, our objective is to predict metabolic syndrome in elderly Chinese individuals at high risk of stroke by utilizing adiposity measures and lipid-related indices.

Waist circumference (WC), hip circumference (HC), and body mass index (BMI) are widely used anthropometric measurements in epidemiological and clinical studies for classifying overweight and obesity [9]. Waist-to-height-ratio (WHtR) and waist-to-hip-ratio (WHR) are considered better indicators of metabolic risk, as they reflect body fat distribution and upper-lower body obesity [10].The conicity index (CI) is based on the observation that individuals with greater fat accumulation in the abdominal region exhibit a biconical shape, while those with less central fat accumulation display a cylindrical shape [11]. Elevated plasma triacylglycerol (TG) and reduced high-density lipoprotein cholesterol (HDL-C) levels have been linked to cardiovascular disease, and their ratio, TG/HDL-C, has been proposed as a novel biomarker for predicting the risk of various clinical conditions [12]. Additionally, several newly developed indices have been introduced for estimating visceral fat.A body shape index (ABSI) expresses the excess risk from high WC in a convenient form that is complementary to BMI and to other known risk factors [13].Body roundness index (BRI), a new shape measure, is a predictor of % body fat and % VAT and can be applied as a visual tool for health status evaluations [14].Visceral adiposity index (VAI) is significantly associated with a range of metabolic diseases, but it is only suitable for Caucasians, while Chinese Visceral Adiposity Index (CVAI) is specifically designed to estimate visceral fat area in Asians [15].Lipid accumulation product (LAP) is emergent predictor of central lipid accumulation linked to diabetes risk and cardiovascular disease [16].Triglycerides and glucose (TyG) index serves as a useful tool for identifying subjects with decreased insulin sensitivity [17].

The optimal approach to using anthropometric indicators for predicting metabolic diseases is still under debate [18]. Therefore, this study aimed to first explore the screening and predictive capabilities of adiposity measures and lipid-related indices for MetS among elderly Chinese individuals at high risk of stroke, with specific emphasis on gender-stratified analyses to account for potential sex-based differences in physiological responses and disease mechanisms. Furthermore, the study sought to identify optimal cut-off values for these indices in both male and female subgroups, laying the groundwork for the prevention and management of MetS.

## Methods

### Study participants

**Fig 1:**
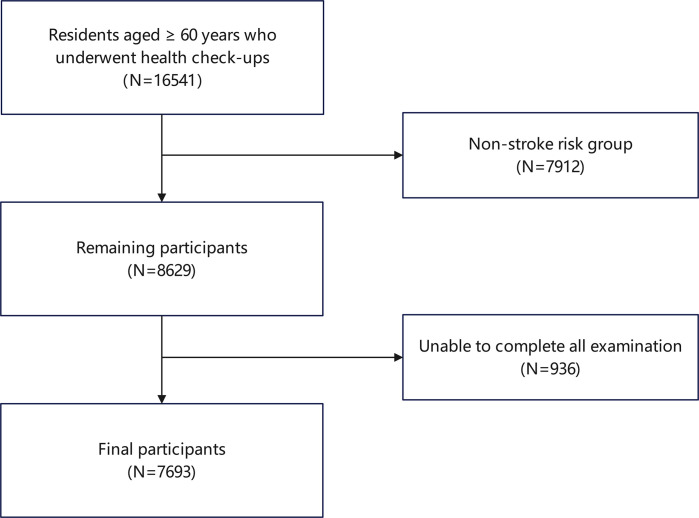
Flowchart for the selection of the analyzed study sample.

This cross-sectional study surveyed senior citizens in Shanghai, conducted within the community. The ethical committee of Zhujing Community Health Service Centre, Jinshan District, Shanghai, China, approved the study at December 12th, 2023 (zjsqtjxm202312001). Physical examination data were collected from 16,451 residents aged 60 years and older who underwent health check-ups at the community health center in Zhujing Community in 2023. We accessed the data on December 5, 2024. The authors did not have access to personally identifiable information about the participant during or after data collection. Ultimately, a total of 7,693 Chinese elderly subjects (3,324 males and 4,369 females) with complete data were included in the study. The participant flowchart is shown in Fig 1.

### Data collection

All medical examination staff underwent standardized training. For all subjects, data were collected on sex, age, blood pressure, height, waist circumference, hip circumference, weight, current smoking status, and alcohol consumption. The waist height ratio (WHtR) was calculated as the ratio of waist circumference to height, and the waist hip ratio (WHR) was determined as the ratio of waist circumference to hip circumference. After a 12-hour fast, peripheral venous blood samples (5 ml) were collected from all residents undergoing physical examinations. Fasting blood glucose, total cholesterol, triglycerides, high-density lipoprotein cholesterol (HDL-C), and low-density lipoprotein cholesterol (LDL-C) levels were measured using a Roche biochemistry analyzer.

The adiposity measures and lipid-related indices included BMI, WC, HC, WHtR, WHR, TG/HDL-C, LAP, VAI, CVAI, ABSI, BRI, CI, TyG, triglyceride glucose-body mass index (TyG-BMI), triglyceride glucose-waist circumference (TyG-WC), triglyceride glucose-hip circumference (TyG-HC), triglyceride glucose-waist-to-height-ratio (TyG-WHtR) and triglyceride glucose-waist-to-hip-ratio (TyG-WHR). These indicators were calculated using the following equations [13–16] 19–24]:


$$\begin{array}{c} \mathrm{\backslash [}BMI\;=\;Weight\;/\;Height² \end{array}$$

WHtR = WC / Height\]

WHR = WC / HC\]

TG/HDL−C = TG / HDL−C\]

LAP (Males) = (WC(cm) − 65) × TG\]

LAP (Females) = (WC(cm) − 58) × TG\]

VAI (Males) = WC(cm)/ (39.68 + (1.88 × BMI)) × (TG(mmol/L) / 1.03) × (1.31 / HDL(mmol/L)) \]

VAI (Females) = WC(cm)/ (36.58 + (1.89 × BMI)) × (TG(mmol/L)/ 0.81) × (1.52 / HDL(mmol/L)) \]

$$\begin{array}{cc} CVAI\;(Males)\; & =\;-267.93\;+\;0.68\;\times \;Age\;+\;0.03\;\times \;BMI\;+\;4.00\;\times \;WC(cm)\;\\ & \;\;\;\;+\;22.00\;\times \;log10(TG(mmol/L))\;-\;16.32\;\times \;HDL(mmol/L)\; \end{array}$$

$$\begin{array}{cc} CVAI\;(Females)\; & =\;-187.32\;+\;1.71\;\times \;Age\;+\;4.23\;\times \;BMI\;+\;1.12\;\times \;WC(cm)\;\\ & \;\;\;\;\;+\;39.76\;\times \;log10(TG(mmol/L))\;-\;11.66\;\times \;HDL(mmol/L) \end{array}$$

$$ABSI\;=\;WC\;/\;({Height}^{1/2}\times \;{BMI}^{2/3})\mathrm{\backslash ]}$$

$$BRI\;=\;364.2\;-\;365.5\;\times \sqrt{1-(WC/{\left(2x\right)}^{2}/\;{\left(0.5\times Height\right)}^{2}}\mathrm{\backslash ]}$$

$$CI\;=\;WC\;(m)\;/\;(0.019\;\times \sqrt{Weight\;(kg)/Height(m)})\mathrm{\backslash ]}$$

TyG index = Ln[(TG(mg/dl) × Glucose(mg/dl)) / 2]\]

TyG−BMI = TyG × BMI\]

TyG−WC = TyG × WC\]

TyG−HC = TyG × HC\]

TyG−WHtR = TyG × WHtR\]

TyG−WHR = TyG × WHR\]

### Definition of MetS

MetS is defined according to the Chinese Guidelines for the Prevention and Treatment of Type 2 Diabetes (2020 edition) [25]. A diagnosis is made when three or more of the following criteria are present.

1. Abdominal obesity (central obesity) is defined as a waist circumference of ≥ 90 cm for men and ≥ 85 cm for women.
2. Hyperglycemia is defined as fasting blood glucose levels of ≥ 6.1 mmol/L or 2-hour post-glycemic load blood glucose levels of ≥ 7.8 mmol/L. This condition also includes individuals who have been diagnosed with and are receiving treatment for diabetes mellitus.
3. Hypertension is defined as a blood pressure of ≥ 130/85 mmHg and/or includes individuals who have been diagnosed with and are being treated for hypertension.
4. Fasting triglycerides of ≥ 1.70 mmol/L.
5. Fasting HDL-C levels below 1.04 mmol/L.

### Definition of high risk groups for stroke

High risk groups for stroke are defined according to the Chinese Guidelines for the Technical Specifications for Stroke Screening and Prevention [26]. For individuals over 40 years of age, stroke risk screening is evaluated based on the following eight risk factors (1 point for each risk factor).

1. A history of hypertension (≥ 140/90 mmHg) or the use of antihypertensive medication.
2. Heart diseases, including atrial fibrillation and heart valve disease.
3. Smoking.
4. Dyslipidemia.
5. Diabetes.
6. Little physical activity.
7. People with overweight or obesity (BMI ≥ 26 kg/m²).
8. Family history of stroke.

Individuals with a stroke risk assessment score of 3 or higher, or a prior history of ischemic stroke or TIA.

### Statistical analysis

Statistical analysis was conducted using R version 4.4.2 and IBM SPSS Statistical Software, version 30 (IBM Corporation, Armonk, New York, NY, USA). Categorical variables were expressed as percentages (%), and results were calculated using the χ² test. Normality was assessed using the Kolmogorov-Smirnov test. Normally distributed variables were analyzed using the t-test, with results presented as mean ± standard deviation. For variables with skewed distributions, results were described by the median and interquartile range and analyzed using the Mann–Whitney U test.

We assessed the degree of correlation between covariates and adiposity measures, lipid-related indices, and sex, as presented in Table 1. In S1 Table, we examined the association between these indices and the presence or absence of MetS. A receiver operating characteristic (ROC) curve was generated, and the area under the curve (AUC) was calculated to evaluate the ability of these indices to identify MetS. Cutoff points were determined using the Youden index (sensitivity + specificity – 1). We calculated accuracy, sensitivity, specificity, positive predictive value (PPV), and negative predictive value (NPV) for each metric to identify predictors.

**Table 1:** Baseline characteristics of the study population.

Variable	Total (n = 7693)	Male(n = 3324)	Female(n = 4369)	P value
Mets				
With	3547 (46.11)	1436 (43.20)	2111 (48.32)	<0.001
Without	4146 (53.89)	1888 (56.80)	2258 (51.68)	
Current smoking				
No	6401 (83.21)	2032 (61.13)	4369 (100.00)	<0.001
Yes	1292 (16.79)	1292 (38.87)	0 (0.00)	
Alcohol drinking				
No	6561 (85.29)	2192 (65.94)	4369 (100.00)	<0.001
Yes	1132 (14.71)	1132 (34.06)	0 (0.00)	
Age (years)	69 (65, 74)	69 (65,74)	69 (65,74)	0.010
BMI (kg/m2)	25.35 (23.38, 27.59)	25.28 (23.38, 27.34)	25.44 (23.39, 27.78)	0.006
WC (cm)	86 (80, 91)	87 (82, 92)	85 (79, 90)	<0.001
HC (cm)	94.00 (90.00, 99.00)	95 (91, 99)	94 (89, 98)	<0.001
WHtR	0.54 (0.51, 0.58)	0.53 (0.50, 0.56)	0.55 (0.52, 0.59)	<0.001
WHR	0.91 (0.87, 0.95)	0.91 (0.88, 0.95)	0.90 (0.86, 0.95)	<0.001
TG/HDL-C	1.03 (0.69, 1.64)	1.06 (0.69, 1.72)	1.02 (0.69, 1.59)	0.055
LAP	35.36 (22.00, 54.81)	30.36 (17.85, 48.55)	39.40 (25.90, 59.20)	<0.001
VAI	1.67 (1.07, 2.66)	1.34 (0.87, 2.18)	1.91 (1.27, 2.99)	<0.001
CVAI	121.94 (99.33, 143.84)	114.92 (88.52, 141.65)	125.62 (105.92, 144.92)	<0.001
ABSI	0.79 (0.76,0.82)	0.78 (0.76,0.81)	0.79 (0.76,0.82)	<0.001
BRI	5.08 (4.53, 5.72)	4.65 (4.25, 5.11)	5.47 (4.92, 6.07)	<0.001
CI	1.24 (1.19, 1.29)	1.23 (1.19, 1.28)	1.24 (1.19, 1.30)	<0.001
TyG	8.89 (8.53, 9.28)	8.85 (8.47, 9.28)	8.91 (8.57, 9.28)	<0.001
TyG-BMI	226.91 (204.45, 251.24)	225.77 (202.80, 249.39)	227.82 (205.45, 252.97)	<0.001
TyG-WC	765.28 (700.34, 832.99)	774.96 (706.58, 842.72)	758.23 (696.03, 824.78)	<0.001
TyG-HC	840.19 (782.52,901.94)	845.77 (785.18,909.57)	836.01 (780.30,896.99)	<0.001
TyG -WHtR	4.84 (4.42, 5.29)	4.70 (4.30, 5.11)	4.95 (4.54, 5.42)	<0.001
TyG -WHR	8.09 (7.55, 8.64)	8.11 (7.59, 8.65)	8.07 (7.52, 8.63)	0.063

Categorical variables were expressed as percentages (%), and results were calculated using the χ² test. Normally distributed variables were analyzed using the t-test, with results presented as mean ± standard deviation. For variables with skewed distributions, results were described by the median and interquartile range and analyzed using the Mann–Whitney U test.

MetS, metabolic syndrome; BMI,body mass index; WC, waist circumference; HC, hip circumference; WHtR, waist-to-height ratio; WHR, waist-to-hip ratio; TG/HDL-C, triglyceride/high-density lipoprotein cholesterol; LAP, lipid accumulation product; VAI, visceral adiposity index; CVAI, Chinese visceral adiposity index; ABSI, a body shape index; BRI, body roundness index; CI, conicity index; TyG, triglyceride glucose index.

Based on the optimal cutoff values of the adiposity measures and lipid-related indices, we categorized them into two variables. The odds ratio (OR) and 95% confidence interval (95% CI) for each adiposity measure and lipid-related index associated with MetS were recalculated. After adjusting for age, current smoking, and alcohol consumption, the OR and 95% CI for each adiposity measure and lipid-related index related to MetS were computed.

## Results

The baseline characteristics of the study population are summarized in Table 1. A total of 7,693 participants were included in this study, comprising 3,324 males (43.2%) and 4,369 females (56.8%). Significant differences were observed between men and women in terms of MetS, smoking habits, alcohol consumption, age, height, weight, BMI, WC, HC, WHtR, WHR, LAP, VAI, CVAI, ABSI, BRI, CI, TyG, TyG-BMI, TyG-WC, TyG-HC, and TyG-WHtR (all P < 0.05). However, TG/HDL-C and TyG-WHR were not statistically significant between the male and female subgroups (P > 0.05). Given these notable distinctions, separate analyses were conducted based on sex.

The baseline characteristics of the study participants, categorized by sex and the presence of MetS, are summarized in S1 Table. The prevalence of MetS is significantly higher among women (48.32%, 95%CI,46.83%, 49.81%) than among men (43.20%, 95%CI, 41.52%, 44.88%). Except for smoking and alcohol consumption, significant differences were observed in the remaining indicators between men with and without MetS. In contrast, significant differences were found across all indicators among women, except for age.

**Fig 2:**
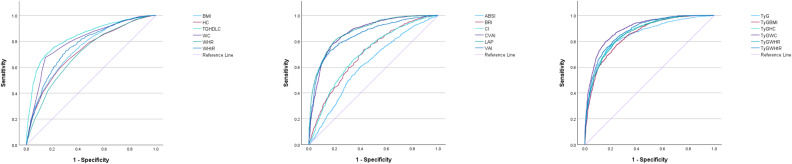
The ROC curves of each indicator in the prediction of MetS in males.

**Fig 3:**
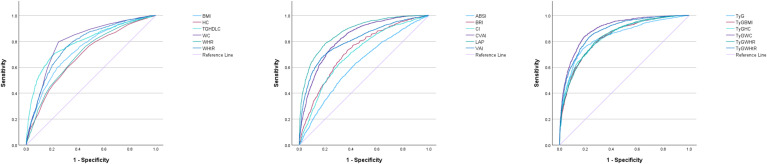
The ROC curves of each indicator in the prediction of MetS in females.

The results of ROC analysis and AUC values for adiposity measures and lipid-related indices are summarized in Table 2. Figs 2 and 3 depict the ROC curves for each indicator in predicting MetS among both men and women. As shown in the graphs and tables, among men, TyG-WC demonstrates the highest predictive value for identifying MetS in the elderly male population (AUC = 0.90, optimal cut-off = 790.629). Other indicators with high predictive values include LAP (AUC = 0.88, optimal cut-off = 33.23), TyG-WHtR (AUC = 0.88, optimal cut-off = 4.753), TyG-HC (AUC = 0.87, optimal cut-off = 864.122), and CVAI (AUC = 0.87, optimal cut-off = 121.764). These indices exhibit similar predictive capabilities. Among women, TyG-WC remains the most predictive indicator (AUC = 0.90, optimal cut-off = 758.02), followed closely by TyG-WHtR (AUC = 0.88, optimal cut-off = 4.918) and LAP (AUC = 0.87, optimal cut-off = 40.835), which also show comparable predictive values. In contrast, the predictive power of ABSI is notably poor in both males and females, indicating its limited utility in identifying MetS.

**Table 2:** The AUCs, optimal cut-off values, sensitivity, specificity, PPV, NPV and Youden index of the 18 parameters for predicting MetS.

Variable	AUC (95% CI)	Cut-off value	Accuracy	Sensitivity	Specificity	PPV	NPV	Youden index
**Male**								
BMI (kg/m2)	0.75 (0.73-0.76)	25.478	0.68	0.69	0.67	0.73	0.62	0.36
WC (cm)	0.80 (0.79-0.82)	89.5	0.78	0.85	0.68	0.78	0.78	0.53
HC (cm)	0.72 (0.71-0.74)	96.5	0.68	0.74	0.59	0.71	0.64	0.33
WHtR	0.77 (0.75-0.78)	0.528	0.71	0.69	0.73	0.77	0.64	0.42
WHR	0.71 (0.69-0.72)	0.905	0.64	0.58	0.72	0.73	0.57	0.30
TG/HDL-C	0.84 (0.82-0.85)	1.289	0.78	0.85	0.70	0.79	0.78	0.55
LAP	0.88 (0.87-0.89)	33.23	0.80	0.81	0.80	0.84	0.76	0.61
VAI	0.85 (0.83-0.86)	1.579	0.79	0.83	0.73	0.80	0.77	0.56
CVAI	0.87 (0.85-0.88)	121.764	0.80	0.82	0.77	0.83	0.77	0.59
ABSI	0.62 (0.60-0.64)	0.786	0.60	0.60	0.60	0.67	0.53	0.20
BRI	0.70 (0.68-0.72)	4.536	0.64	0.58	0.73	0.74	0.57	0.31
CI	0.70 (0.68-0.72)	1.238	0.66	0.65	0.66	0.72	0.59	0.31
TyG	0.85 (0.83-0.86)	8.91	0.78	0.78	0.78	0.82	0.73	0.56
TyG-BMI	0.85 (0.84-0.86)	228.344	0.76	0.76	0.77	0.81	0.71	0.53
TyG-WC	0.90 (0.89-0.91)	790.629	0.82	0.84	0.80	0.85	0.79	0.64
TyG-HC	0.87 (0.85-0.88)	864.122	0.79	0.82	0.75	0.81	0.76	0.57
TyG -WHtR	0.88 (0.87-0.89)	4.753	0.79	0.79	0.80	0.84	0.74	0.59
TyG -WHR	0.86 (0.85-0.87)	8.137	0.78	0.76	0.81	0.84	0.72	0.57
**Female**								
BMI (kg/m2)	0.74 (0.72-0.75)	25.922	0.68	0.74	0.62	0.68	0.69	0.36
WC (cm)	0.80 (0.79-0.81)	84.5	0.77	0.75	0.80	0.80	0.75	0.55
HC (cm)	0.69 (0.67-0.71)	94.5	0.64	0.68	0.60	0.64	0.64	0.28
WHtR	0.78 (0.76-0.79)	0.552	0.72	0.70	0.74	0.74	0.70	0.44
WHR	0.70 (0.69-0.72)	0.885	0.65	0.54	0.76	0.71	0.61	0.30
TG/HDL-C	0.79 (0.78-0.81)	1.137	0.75	0.80	0.69	0.73	0.76	0.49
LAP	0.87 (0.86-0.88)	40.835	0.79	0.80	0.78	0.80	0.79	0.58
VAI	0.81 (0.79-0.82)	2.199	0.75	0.83	0.67	0.73	0.79	0.50
CVAI	0.83 (0.81-0.84)	126.734	0.75	0.76	0.75	0.76	0.74	0.51
ABSI	0.62 (0.61-0.64)	0.786	0.60	0.56	0.63	0.62	0.57	0.19
BRI	0.73 (0.71-0.74)	5.37	0.68	0.63	0.73	0.71	0.65	0.36
CI	0.71 (0.69-0.72)	1.251	0.66	0.70	0.62	0.66	0.66	0.32
TyG	0.84 (0.82-0.85)	8.97	0.78	0.82	0.74	0.77	0.79	0.56
TyG-BMI	0.83 (0.82-0.85)	222.714	0.75	0.69	0.82	0.81	0.71	0.51
TyG-WC	0.90 (0.89-0.90)	758.02	0.82	0.81	0.84	0.84	0.81	0.65
TyG-HC	0.84 (0.83-0.85)	834.247	0.75	0.74	0.77	0.78	0.73	0.51
TyG -WHtR	0.88 (0.87-0.89)	4.918	0.80	0.77	0.83	0.83	0.77	0.60
TyG -WHR	0.84 (0.83-0.85)	8.025	0.76	0.73	0.79	0.79	0.73	0.52

BMI,body mass index; WC, waist circumference; HC, hip circumference; WHtR, waist-to-height ratio; WHR, waist-to-hip ratio; TG/HDL-C, triglyceride/high-density lipoprotein cholesterol; LAP, lipid accumulation product; VAI, visceral adiposity index; CVAI, Chinese visceral adiposity index; ABSI, a body shape index; BRI, body roundness index; CI, conicity index; TyG, triglyceride glucose index.PPV, positive predictive value; NPV, negative predictive value.

**Fig 4:**
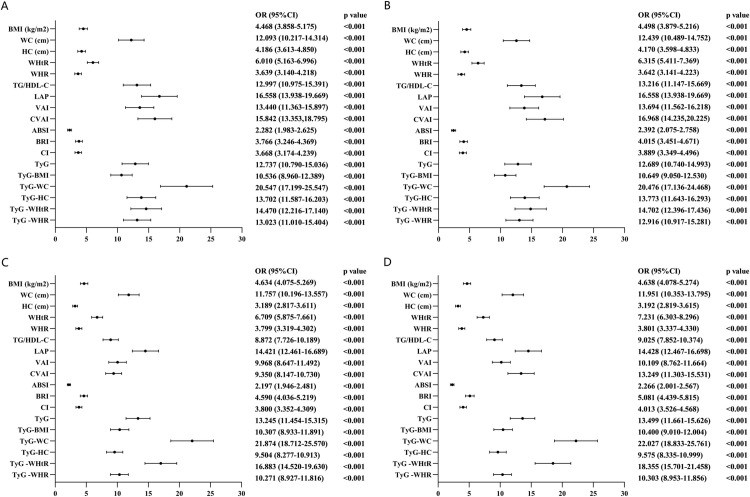
Forest diagram of OR before and after adjustment of confounding factors. **(A)** Male unadjusted **(B)** Male adjusted **(C)** Female unadjusted **(D)** Female adjusted Adjusted OR:Adjusted for age, current smoking, alcohol drinking.

Based on the cut-off values presented in Table 2, the adiposity measures and lipid-related indices were categorized into binary variables for this study. Given that age, smoking, and alcohol consumption have been established as contributing factors to MetS [27–29], these variables were included as potential confounders. Forest plots depicting the OR for both males and females, before and after adjustment for confounders, are shown in Fig 4. For example, in men, each unit increase in TyG-WC is associated with a 20.476-fold increase in the likelihood of developing MetS. Similarly, in women, each unit increase in TyG-WHtR is associated with an 18.355-fold increase in the likelihood of developing MetS.

## Discussion

This study evaluated the predictive capabilities of various adiposity measures and lipid-related indices for MetS among elderly Chinese individuals at high risk of stroke. The findings revealed that the TyG index and its associated metrics are highly effective in predicting MetS. Specifically, TyG-WC demonstrated the highest predictive performance, followed by TyG-WHtR and LAP. These results highlight the importance of combining lipid indices with anthropometric measures to enhance MetS identification in high-risk elderly populations.

The superior predictive performance of TyG-WC, TyG-WHtR and other TyG-related indices can be attributed to their ability to integrate information on both lipid metabolism and body fat distribution. The TyG index is a crucial tool for predicting and diagnosing outcomes related to insulin resistance in clinical populations [30]. By combining the TyG index with anthropometric features such as WC, WHtR, and HC, we can better capture the complex interplay between adiposity and metabolic dysfunction, thereby improving the accuracy of MetS prediction. Among a range of obesity indices, TyG-WHtR and TyG-WC are the optimal predictors for metabolic syndrome in American adults aged 18–64 [31]and Chinese individuals aged 45 and above [32].Thus, TyG-related indices are expected to be promoted to more populations.

As a novel proxy for central obesity and lipid accumulation, LAP also exhibited robust predictive performance for MetS. It can be used to assess visceral fat distribution and reflect visceral fat dysfunction by integrating anthropometric markers with lipid or glucose markers. A study has indicated that LAP demonstrates significant predictive efficacy for MetS in a relatively healthy Chinese population [33]. A study has also found that LAP demonstrates good predictive ability for MetS in low-income rural adults in Xinjiang, China [34].Therefore, LAP may have the potential to predict MetS in all Chinese population.

In contrast, ABSI showed notably poor predictive performance, with an AUC of 0.62 in both males and females. This may be due to its high concentration around the mean and relatively small variance [35]. The relatively poor performance of ABSI in predicting chronic diseases has been observed in several other studies [32,36].

It is worth noting that several components of the assessed measures, such as BMI, WC, and TG/HDL-C, are already part of the diagnostic criteria for MetS. This likely contributed to the high AUC measurements observed for some of the indices. However, the combination of these components with other lipid and anthropometric measures provided additional predictive value, suggesting that these combined indices may offer more comprehensive insights into MetS risk compared to the individual diagnostic criteria alone.

From a clinical perspective, while the MetS definition is widely used and relatively straightforward to apply, the combined lab and anthropometric measurements studied here may provide more nuanced and accurate assessments of MetS risk. This could be particularly valuable in high-risk populations, such as elderly individuals at risk of stroke, where early and precise identification of MetS is crucial for targeted interventions. However, the feasibility of implementing these more complex measures in clinical settings should be carefully considered, as they may require additional resources and expertise compared to the existing MetS diagnostic criteria.

This study had several limitations. First, as a cross-sectional study, it was unable to establish causal relationships. Second, the participants were exclusively elderly Chinese individuals at high risk of stroke, which limits the generalizability of the findings to other ethnic groups and populations. Third, detailed information regarding participants’ chronic medical histories, long-term medication use, educational attainment, and physical activity levels was not collected. These omissions may have influenced the overall results.

## Conclusion

Among elderly Chinese individuals at high risk of stroke, all adiposity measures and lipid-related indices effectively predicted MetS, with the exception of ABSI, which exhibited poor predictive performance. In both men and women, TyG-WC emerged as the strongest predictor of MetS, followed by TyG-WHtR and LAP.

Next, additional studies should be conducted in more generalizable populations covering different ethnic groups and regions to validate the universal applicability of these measures. Meanwhile, these indices, especially TyG-WC, TyG-WHtR and LAP, can be further explored for integration into clinical workflows or electronic health records to facilitate the early screening and management of MetS in high-risk populations, so as to provide a basis for formulating more targeted prevention and intervention strategies.

## Supporting information

S1 Table Baseline characteristics between the metabolic syndrome (MetS) and non-metabolic syndrome (Non-MetS) groups for each sex. Categorical variables were expressed as percentages (%), and results were calculated using the χ² test. Normally distributed variables were analyzed using the t-test, with results presented as mean ± standard deviation. For variables with skewed distributions, results were described by the median and interquartile range and analyzed using the Mann–Whitney U test. MetS, metabolic syndrome; BMI,body mass index; WC, waist circumference; HC, hip circumference; WHtR, waist-to-height ratio; WHR, waist-to-hip ratio; TG/HDL-C, triglyceride/high-density lipoprotein cholesterol; LAP, lipid accumulation product; VAI, visceral adiposity index; CVAI, Chinese visceral adiposity index; ABSI, a body shape index; BRI, body roundness index; CI, conicity index; TyG, triglyceride glucose index.
(DOCX)

S1 File Raw data. (XLSX)
