# Supplementary material for: Predictive value of adiposity measures and lipid-related indices for metabolic syndrome in Chinese elderly people at high risk of stroke
Source: PLoS One. 2025 Aug 12;20(8):e0328275. doi: 10.1371/journal.pone.0328275 (PMC12342311; doi:10.1371/journal.pone.0328275)
Supplement: S1 Table — Categorical variables were expressed as percentages (%), and results were calculated using the χ² test. Normally distributed variables were analyzed using the t-test, with results presented as mean ± standard deviation. For variables with skewed distributions, results were described by the median and interquartile range and analyzed using the Mann–Whitney U test. MetS, metabolic syndrome; BMI,body mass index; WC, waist circumference; HC, hip circumference; WHtR, waist-to-height ratio; WHR, waist-to-hip ratio; TG/HDL-C, triglyceride/high-density lipoprotein cholesterol; LAP, lipid accumulation product; VAI, visceral adiposity index; CVAI, Chinese visceral adiposity index; ABSI, a body shape index; BRI, body roundness index; CI, conicity index; TyG, triglyceride glucose index. (DOCX) [file pone.0328275.s001.docx]

**S1 Table. Baseline characteristics between the metabolic syndrome (MetS) and non-metabolic syndrome (Non-MetS) groups for each sex.**

| Variable | Male(n=3324) | | | Female(n=4369) | | |
| --- | --- | --- | --- | --- | --- | --- |
|  | MetS(n=1436) | Non-MetS(n=1888) | P value | MetS(n=2111) | Non-MetS(n=2258) | P value |
| Current smoking | 567 (39.48) | 725 (38.40) | 0.525 | 0 | 0 | / |
| Alcohol drinking | 504 (35.10) | 628 (33.26) | 0.269 | 0 | 0 | / |
| Age (years) | 68 (64,73) | 69 (65,74) | <0.001 | 69 (65,74) | 69 (65,74) | 0.224 |
| BMI (kg/m2) | 26.70 (24.86,28.81) | 24.22 (22.48,26.08) | <0.001 | 26.90 (24.78,29.01) | 24.20 (22.31,26.12) | <0.001 |
| WC (cm) | 92 (87,96) | 84 (79,88) | <0.001 | 89 (85,94) | 80 (76,84) | <0.001 |
| HC (cm) | 98 (94,102) | 93 (90,97) | <0.001 | 96 (92,100) | 91 (87,96) | <0.001 |
| WHtR | 0.55 (0.53,0.58) | 0.51 (0.48,0.54) | <0.001 | 0.58 (0.55,0.62) | 0.53 (0.50,0.56) | <0.001 |
| WHR | 0.94 (0.90,0.97) | 0.90 (0.86,0.93) | <0.001 | 0.93 (0.89,0.97) | 0.88 (0.84,0.92) | <0.001 |
| TG/HDL-C | 1.72 (1.17,2.45) | 0.81 (0.56,1.10) | <0.001 | 1.47 (0.99,2.22) | 0.80 (0.57,1.05) | <0.001 |
| LAP | 49.21 (35.36,69.74) | 20.64 (12.92,30.14) | <0.001 | 57.50 (42.39,80.61) | 27.59 (19.24,37.99) | <0.001 |
| VAI | 2.20 (1.50,3.14) | 1.00 (0.68,1.38) | <0.001 | 2.78 (1.89,4.20) | 1.47 (1.05,1.96) | <0.001 |
| CVAI | 140.47 (123.46,159.08) | 95.43 (71.86,114.74) | <0.001 | 140.60 (126.49,158.23) | 109.75 (94.06,126.20) | <0.001 |
| ABSI | 0.80 (0.77,0.82) | 0.78 (0.75,0.81) | <0.001 | 0.80 (0.77,0.83) | 0.78 (0.75,0.81) | <0.001 |
| BRI | 4.90 (4.52,5.32) | 4.46 (4.10,4.90) | <0.001 | 5.81 (5.32,6.35) | 5.15 (4.68,5.69) | <0.001 |
| CI | 1.26±0.06 | 1.21±0.07 | <0.001 | 1.27 (1.22,1.32) | 1.22 (1.17,1.27) | <0.001 |
| TyG | 9.27 (8.96,9.63) | 8.59 (8.30,8.86) | <0.001 | 9.23 (8.96,9.60) | 8.65 (8.41,8.89) | <0.001 |
| TyG-BMI | 248.57 (229.48,268.05) | 209.31 (190.78,227.84) | <0.001 | 248.82 (228.83,270.09) | 209.89 (193.67,228.48) | <0.001 |
| TyG-WC | 852.10±76.46 | 717.76±78.34 | <0.001 | 818.42 (775.55,875.55) | 703.52 (655.36,744.61) | <0.001 |
| TyG-HC | 908.69 (861.99,956.97) | 801.29 (754.28,846.96) | <0.001 | 887.76 (838.37,944.54) | 791.38 (750.12,836.75) | <0.001 |
| TyG -WHtR | 5.15±0.48 | 4.38±0.48 | <0.001 | 5.37 (5.04,5.75) | 4.60 (4.27,4.88) | <0.001 |
| TyG -WHR | 8.65 (8.23,9.10) | 7.74 (7.29,8.12) | <0.001 | 8.55 (8.10,9.08) | 7.62 (7.20,8.07) | <0.001 |

Categorical variables were expressed as percentages (%), and results were calculated using the χ² test. Normally distributed variables were analyzed using the t-test, with results presented as mean ± standard deviation. For variables with skewed distributions, results were described by the median and interquartile range and analyzed using the Mann–Whitney U test.

MetS, metabolic syndrome; BMI,body mass index; WC, waist circumference; HC, hip circumference; WHtR, waist-to-height ratio; WHR, waist-to-hip ratio; TG/HDL-C, triglyceride/high-density lipoprotein cholesterol; LAP, lipid accumulation product; VAI, visceral adiposity index; CVAI, Chinese visceral adiposity index; ABSI, a body shape index; BRI, body roundness index; CI, conicity index; TyG, triglyceride glucose index.
